# Receptiveness and Responsiveness Toward Using Social Media for Safe Firearm Storage Outreach: Mixed Methods Study

**DOI:** 10.2196/24458

**Published:** 2021-06-18

**Authors:** Esther Lam, Megan Moreno, Elizabeth Bennett, Ali Rowhani-Rahbar

**Affiliations:** 1 Department of Epidemiology School of Public Health University of Washington Seattle, WA United States; 2 School of Medicine and Public Health University of Wisconsin Madison, WI United States; 3 Seattle Children's Hospital Seattle, WA United States

**Keywords:** firearm storage, gun safety, public health outreach, social media, mixed methods, family

## Abstract

**Background:**

Childhood and adolescent firearm injury and death rates have increased over the past decade and remain major public health concerns in the United States. Safe firearm storage has proven to be an effective measure to prevent firearm injury and death among youth. Social media has been used as an avenue to promote safe firearm storage, but perceptions of this tool remain unknown.

**Objective:**

The aim of this study was to determine receptiveness and responsiveness in promoting firearm lock box and trigger lock giveaway events on social media, and to describe the characteristics of participants who learned of these events through social media.

**Methods:**

We performed a mixed methods study combining a content analysis of Facebook event post comments, quantitative analysis of positive and negative feedback on social media, and a descriptive analysis of event participant characteristics. Through a qualitative content analysis approach, we thematically coded comments from each event’s social media page posting. Interrater reliability and κ statistics were calculated. We calculated the prevalence of positive and negative feedback data. Further, we calculated descriptive statistics for demographic characteristics gathered from day-of-event intake surveys. Differences between collected measures were analyzed with χ^2^ and *t* tests according to how the participant found out about the event (social media vs other means). Using concurrent analysis, we synthesized the results from both the qualitative and quantitative aims.

**Results:**

Through qualitative content analysis, 414 comments from 13 events were coded. Seven themes emerged through the comment coding process with the most common being “positive receptiveness” (294/414, 71.0%). From quantitative analysis of the social media content, we found higher levels of positive feedback compared to negative feedback. The average number of event post “likes” was 1271.3 per event, whereas the average count in which “hide post” was clicked was 72.3 times per event. Overall, 35.9% (1457/4054) of participants found out about the event through social media. The participants who learned about the event through social media were on average significantly younger than those who learned about the event through other means (–6.4 years, 95% CI –5.5 to –7.3). Among the group that learned of the event through social media, 43.9% (629/1433) identified as female, whereas 35.5% (860/2420) identified as female among the group that learned of the event through other means.

**Conclusions:**

There was overall positive receptiveness and responsiveness toward firearm lock box and trigger lock giveaway events when promoted on social media. Compared with other promotional tools, social media has the ability to reach those who are younger and those who identify as female. Future studies should extend this research to determine whether there is a difference between rural and urban settings, and consider other social media platforms in the analysis.

## Introduction

### Background

Firearm injury is a major public health concern in the United States. Among children under 17 years of age, approximately 1300 die from and 5790 are treated for gunshot wounds each year [1]. Firearm suicide rates among this population have been increasing over the past few years [1,2] along with increasing rural-urban divides [3-5]. Based on a national study of individuals younger than 20 years, the incidence of firearm injury hospitalization was the highest among 15 to 19-year-olds residing in urban settings [4]. However, hospitalization rates were higher in rural areas than in urban areas for those in the 5-9 and 10-14–year age groups [4].

Safe storage of firearms is associated with reduced risk of unintentional and intentional self-inflicted firearm injury among youth [6]. Examples of safe firearm storage practices include storing the firearm in a locked location such as a gun safe or lockbox, or with a trigger lock, and storing it unloaded with the ammunition stored separately [6]. A study conducted in the United States found that up to 65% of all surveyed high school seniors in a midwestern state had access to at least one firearm in their household [7]. An evaluation with a nationally representative sample found that approximately 1 in every 3 adolescents reported living in a home with firearm access [8]. Moreover, 1 in every 5 firearm-owning households with children store their firearms in the least safe manner (ie, loaded and unlocked) [9].

In Washington state, 34% of households reported having a gun at home, and only 38% of those reported storing their guns locked and unloaded [10]. In a survey of 2956 participants at safe firearm storage events in Washington state, 40.1% indicated that they stored at least one firearm unlocked [11].

Washington state passed Initiative Measure No. 1639 in 2018 [12], which requires a semiautomatic rifle purchaser to provide proof of completion of a recognized firearm safety training program that includes secure gun storage education [12]. Additionally, under this initiative, a person who leaves a firearm in a place where a prohibited person (ie, a child) could potentially gain access to the firearm will be charged with community endangerment if the prohibited person gains access to the firearm [12].

### Safe Firearm Storage Giveaway Outreach Events

Prior to the passing of Initiative Measure No. 1639, Seattle Children’s Hospital began the “Safe Firearm Storage Giveaway Outreach Events” (SFSGOE) program. This program sought to reach both urban and rural communities.  The primary mission of these events was to prevent firearm injuries by creating safe household environments for children, their families, and their communities, and provide free firearm lock boxes and trigger locks supplemented with education and device demonstrations. The events were focused on parents living in households with children. This was a community-based effort involving multiple stakeholders, including other hospitals; public health agencies; coalitions aimed at advocating for safe environments for children; and sporting goods stores that sell firearms, lock boxes, and trigger locks. These efforts have proven to be effective in changing behavior related to safe firearm storage practices. An evaluation survey performed 4-6 weeks after the event found that a significantly greater proportion of households with children and adolescents reported having stored and locked their firearms safely [13]. Flyers, newspaper advertisements, radio, television, sharing through word of mouth, and posting on social media were all used to promote these events. Social marketing, designed to influence health behaviors, prevent injuries, and contribute to communities on a large scale, was used as a framework for promotion and messaging [14,15].

Social media offers a versatile and unique tool for implementing a social marketing approach to increase the number of individuals and communities reached. Accordingly, marketing using social media has become a growing trend in many fields. However, there has been little research performed to understand whether or not social media promotion of topics such as firearm use and storage practices is effective in targeting the appropriate audience and yielding acceptance. Skepticism may arise regarding whether social media platforms are the appropriate virtual venues for potentially controversial conversations, which can yield negative reactions. Therefore, the purpose of this study was to understand the individual- and community-level responses toward social media as an educational and marketing tool for promoting safe firearm storage giveaway events. We also sought to evaluate the attitudes and conversations that emerged through the social media marketing event page posts.

### Study Aims

There were three aims of this study: (1) understand the receptiveness of using social media to promote safe firearm storage in the continuum of rural to urban areas; (2) assess the individual-level responsiveness toward the SFSGOE hosted by Seattle Children’s Hospital; and (3) compare the characteristics of event participants who learned of the event via social media with those of participants who learned of the event through other sources.

## Methods

### Study Design

We used a mixed methods design, incorporating both qualitative and quantitative approaches, which allows for gaining a deeper understanding of pragmatic implementation in addition to increasing reproducibility [16]. Furthermore, other recent studies with the aim of understanding the intersection between social media and health behaviors or conditions have also used this mixed methods approach to allow for a comprehensive review of all metrics and data available on the social media platform of interest [17,18].

For this mixed methods approach, we utilized a convergent design. Since both elements of this study pull from existing data, quantitative analysis and qualitative evaluation occurred concurrently, and the results were then merged to supplement each other upon synthesis.

Conceptualized by our research team for the purpose of this study, receptiveness was defined as the attitudes toward the event and responsiveness was defined as the quantifiable interactions on the event page posts.

### Assessing Rurality Versus Urbanity

The United States Department of Agriculture Economic Research Service Rural-Urban Continuum Codes (RUCC) from 2013 were used to categorize the locations of these events [19]. The 2013 edition was the most recent RUCC available at the time of analysis as these codes are updated every 10 years [19]. Codes are assigned to counties, ranging from 1 to 9 based on population size and proximity to a metropolitan area [19]. Urban counties are coded between 1 and 3; code 1 is given to a city in a large-sized urban county, code 2 is given to a city in a medium-sized urban county, and code 3 is given to a city in a small semiurban county [19]. Nonmetropolitan counties are coded from 4 to 9, with 4 being a city in a larger semirural county and 9 being a city in a small completely rural county [19]. Table 1 lists the SFSGOE locations and their assigned RUCCs.

**Table 1:** Locations and times of events that were evaluated along with their rural and urban codes (RUCC).

Month and year of event	City	County	RUCC
December, 2014	Seattle	King	1
January, 2015	Fife	Pierce	1
April, 2015	Kennewick (Tri-Cities)	Franklin	2
June, 2015	Monroe	Snohomish	1
October, 2015	Tacoma	Pierce	1
November, 2015	Kirkland	King	1
June, 2016	Toppenish	Yakima	3
July, 2016	Marysville	Snohomish	1
October, 2016	Wenatchee	Chelan	3
February, 2017	Seattle	King	1
May, 2017	Mount Vernon	Skagit	3
June, 2017	Lacey	Thurston	2
October, 2017	Moses Lake	Grant	5
March, 2018	Silverdale	Kitsap	2

### Aim 1: Content Analysis to Assess Receptiveness

#### Overview of Content Analysis

Content analysis was used to evaluate comments generated by Facebook users in response to posts advertising and promoting SFSGOE. For this social media content analysis, a codebook was iteratively created to capture the themes in attitudes and perceptions toward these events presented in the coded comments.

#### Data Source

We used data from the social media platform, Facebook, for this analysis because Facebook was the primary platform used for promoting the SFSGOE. Seattle Children’s Hospital created a Facebook event page, posting details of each event and also shared the event on their main Facebook page. The page’s visibility was dependent on the sharing of the event and set reminders innate to Facebook event pages to provide additional promotion.

#### Data Query

A Facebook event page post query was performed. We searched the Seattle Children’s Hospital Facebook posts sharing information about the event and for the Facebook event page. Only comments and metrics linked to the event page that were from Seattle Children’s Hospital shared posts were used in the analysis. Upon searching for these retrospective posts, the study team did not interact (ie, like, share, comment) with the posts; all data provided for this analysis were strictly independent of the study team and unaltered.

#### Data Collection

Data from December 2014 to March 2018 were used for this study. This period included the first event for which Facebook was used to promote the SFSGOE until the most recent Facebook-promoted event at the time of this analysis. All comments linked to the event page posting were deidentified and saved in a password-protected Microsoft Excel spreadsheet. Data from these social media comments were then qualitatively evaluated via exploratory content analysis to understand receptiveness and were quantitatively evaluated to understand responsiveness.

#### Codebook Development and Coding Validation

An iterative categorization approach was used for this analysis to allow for inductive coding, resulting in a structured system that would allow for reproducible results [20]. Using this inductive coding approach, parent and child codes gradually emerged and were created. Parent codes were the overarching salient themes and child codes stemmed from each of the parent codes, with much more specific attitudes and perspectives [21]. Owing to the finite quantity of comments available for this content analysis, we chose to code all available data to ensure that the themes generated from the sampling would be representative. Considering this to be an exploratory content analysis, we aimed to use the inductive thematic saturation model [22]. Since we did not use a priori conceptualized categories or an existing codebook, this model would allow for generation of themes found to be most appropriate to the generated responses from SFSGOE Facebook event pages [22].

At the time of this analysis, there was no known validated assessment for emoticons; therefore, emoticons were not coded. Only comments in the English language were coded. Each comment was only allowed one code; therefore, for lengthier comments, the most explicit and apparent theme was coded. The comment and the assigned code were recorded in a password-protected Microsoft Excel spreadsheet.

All comments, including replies and comments from Seattle Children’s Hospital, were coded. Initial coding was performed by one study team member with prior training in qualitative research methods. Texts from the comments were recorded verbatim, excluding identifiers such as the name of the Facebook user or names of any tagged users.

To evaluate the coding process and to ensure the validity of the codebook, interrater reliability and the κ statistic were calculated. Another study team member with prior training in content analysis, and specifically with this codebook, was given 10% (n= 42) of the comments to code independently. Coding results from this research team member and the first set of coding results were compared. The results indicated a strong level of agreement (κ=0.87, *P*<.001) supporting the reliability of the coding procedures.

### Aim 2: Quantitative Measures and Responsiveness Analysis

Each event allowed for “liking” of the post in addition to commenting. Post “likes” are publicly available data. In addition to these data, we used the SimplyMeasured software system, which tracks deidentified data, including the number of clicks and the “total reach” of the event page post. The “total reach” was defined as the total number of unique individuals who saw the post during the “reporting period.” The “reporting period” was the duration when the post was publicly available. We also qualified the data system in terms of “negative feedback.” Extracted “negative feedback” data displayed the number of individuals who selectively chose to hide the post, report the post as spam, hide all future posts from the same posting account, and those who unliked the page of the account that posted these events.

For each event, prevalence was calculated using the count of each measure as the numerator and the “total reach” number as the denominator.

### Aim 3: Survey

#### Data Collection, Measures, and Inclusion Criteria

At each SFSGOE, as part of the intake and prior to receipt of a trigger lock or lock box, each household was given a paper survey to complete. This survey included demographic questions, current firearm practice questions, questions related to how the household found out about the event, and their intention to use the giveaway device in the next week. Only those who identified as 18 years of age or older were provided the equipment. No questions on the survey were mandatory. An individual’s receipt of a safe firearm storage device was not dependent on completion of the survey. The submission of a survey and release form were used as a proxy to quantify the attendance of these events. The outreach event staff then entered the survey data into a secure database in REDCap [23,24]. Data from the database were then extracted and quantitatively analyzed. Only individual-level responses deemed to be “complete” by the data importer were used.

#### Statistical Analysis

The χ^2^ test was used to examine whether there was a significant difference in distribution of the categorical variables (gender, firearm storage practices, and intention to use in the next week) based on how the individual found out about the event (social media vs other means). The *t* test was used to examine if there was a significant difference in mean age according to how individuals found out about the event. A *P* value less than .05 was used as the cutoff for significance.

### Ethical Approval

The content analysis portion of this study was exempt from Institutional Review Board review because it only used publicly accessible and deidentified data from Facebook. The survey used in this study was approved by the Institutional Review Board of Seattle Children’s Hospital, Seattle, Washington.

## Results

### Aim 1: Content Analysis

A total of 418 comments from 13 event page posts were publicly available during the timeframe of interest. A total of 414 comments were coded; 4 comments were not evaluated because they only used emoticons or were not written in English. From the 15 events that occurred within the timeframe, only 13 resulted in publicly available posts during the data search and extraction. On average, event posts had 32 comments (range 0-131). Of the 13 event page posts evaluated, eight were from areas that were coded as a city in a large urban county (RUCC=1), one was categorized as a city in a medium-sized urban city (RUCC=2), three were categorized as a city in a small-sized semiurban county (RUCC=3), and one was categorized as a city in a medium-sized semirural county (RUCC=5).

A total of seven parent codes were developed during the iterative coding process: (1) Positive Receptiveness, (2) Negative Receptiveness, (3) Postevent Commenting, (4) Advocacy Against Firearm Storage and/or Firearm Control, (5) Advocacy for Firearm Storage and/or Firearm Control, (6) Commenting to Moderate, and (7) Comments by the Host. Stemming from these parent codes, 21 child codes were created. The coding schemas at the end of the inductive coding process along with representative examples of comments are presented in Textbox 1.

The most common parent code was Positive Receptiveness, accounting for 71.0% (294/414) of all comments coded. Positive Receptiveness is defined as a comment showing a positive attitude toward the event, including expressing excitement, gratitude, or interest explicitly, or sharing the event by tagging friends. Expressing excitement or gratitude toward the event accounted for 10.9% (45/414) of all comments. Of all comments coded, 40.3% (167/414) involved Facebook users tagging other Facebook users, which may be an implicit notion of sharing the event post. Among all codes, 6.3% (26/414) of all comments were coded as the Facebook user showing interest for the event, but could not make it to the event due to the distance and timing of the event.

Negative Receptiveness made up 3.6% (15/414) of all comments. Of all comments, 2.9% (12/414) involved individuals expressing that they did not understand why these events were necessary or that they did not think that lock boxes work. Overall, 0.7% (3/414) of individuals expressed doubtful sentiments about the potential success of the event. Postevent commenting made up 0.7% (3/414) of all codes. This included participants going back to the event post and commenting about their experience at the event. Most of these comments were factual experiences, including how long the wait was to receive their giveaway device or what type of firearm storage device they received at the event.

Advocacy Against Firearm Storage and/or Firearm Control made up 3.9% (16/414) of total comments. These comments included sentiments that children should be trained to use firearms and that there should be much more liberal firearm ownership. Advocacy for Firearm Storage and/or Firearm Control made up 7.0% (29/414) of the coded comments. These comments included perspectives that children should be well protected from firearms and there also were some comments that expressed sentiments of wanting more firearm control.

The parent code Commenting to Moderate was used to denote a case in which a Facebook user commented during or subsequent to a political or controversial discussion. We defined this as moderating, as this type of comment would aim at drawing attention back to the goal of the event, alleviating the tone created by controversial comments; 2.2% (9/414) of the coded comments were coded as such. This emergence of commenting to moderate follows with the emergence of “moderators.” For the purpose of this study, a moderator was defined as a Facebook user, not associated with Seattle Children’s Hospital, who facilitates the conversation and draws other Facebook users back to the goal of the event (ie, to promote safe firearm storage practices). These moderating comments were not from the Seattle Children’s Hospital account and there were no known intentions from the hospital to have users take up this role. This was a role that emerged naturally through discussions from Facebook user interactions on the event page posts.

Comments by the Host as a parent code was defined as any comment posted by Seattle Children’s Hospital to answer any inquiries from a comment or to thank commenting users for their support in some cases; 9.4% (39/414) of all comments were Comments by the Host.

Comments that were not relevant to the topic of the event were not coded with any parent or child code and were marked as Spam (2.2%, 9/414).

**Inductively created codebook and representative comments from evaluation of event page posts.**

**Positive Receptiveness**

Any comment showing a positive attitude toward the event, including expressing excitement, gratitude, or interest explicitly or sharing the event by tagging friends.

- Sharing the event by tagging another Facebook user
- Asking for more of these events or suggesting other locations for these events
- Showing appreciation/gratitude toward Seattle Children’s Hospital for hosting these events
- Showing excitement for the event, positive exclamation, or emotion for the event (eg, “Yay!” “Awesome!”)
- Calling for others to go to the event by sharing more details about the event
- Expressing interest for the event but also saying that they cannot go to the event
- Saying that they will go to the event
- Positive comments about lock boxes or trigger locks
- Asking for more information about the event

Example: “Thank you Seattle Children’s and Outdoor Emporium. This was just what my family needed” [Facebook user]

**Negative Receptiveness**

Any comment showing a negative attitude toward the event, including expressing doubt for either the event or a safe firearm storage device

- Does not understand why these events are necessary, or why lock boxes or trigger locks are necessary
- Doubtful about how these events will be successful
- Shared past negative experience with a lock box or trigger lock

Example: “This is a nice give away but I doubt there will be enough boxes...there will be plenty of locks. Every gun has a lock sold with it. And will never be used by anyone with brains” [Facebook user]

**Postevent Commenting**

Any comment shared after the event by an event participant and comments related to their experience at the event

- Shared facts about experience at the event without a sense of positivity or negativity

Example: “After they ran out of Bulldog brand they started handing out Fortress brand lockboxes” [Facebook user]

**Advocacy Against Firearm Storage and/or Firearm Control**

Any comment displaying opposition against safe firearm storage (including lock boxes and trigger locks)

- Shared a story anecdotally or factually that children can handle guns well, or advocating that children learn how to handle guns
- Words of disagreement or discourse against firearm safety
- Advocating for liberal gun ownership

Example: “Problem is in a home invasion you don’t have time to unlock your safe gun box. You lose, that’s what happened to my brother” [Facebook user]

**Advocacy for Firearm Storage and/or Firearm Control**

Any comment displaying support for safe firearm storage (including lock boxes and trigger locks)

- Advocating that children should be well protected from guns
- Words of agreement or discourse relating to firearm safety
- Bringing up banning gun ownership

Example: “But does every other kid who comes in your home? I'm all for firearm education, I went through it all when I was a kid, but fewer kids these days are learning from real educators and more from cartoons and movies. Not trying to pick a fight, just posing a question. My 4 year old play shoots (much against my wishes) and my almost 2 year old has picked up on it. The older one comprehends the severity of death, but the baby has no idea. Lock boxes and trigger locks isn’t to protect the ones who understand, it’s to protect the ones who don’t” [Facebook user]

**Commenting to Moderate**

Any comment from a Facebook user that draws attention back to the mission of the goal of the event

- Moderates a heated discussion and draws attention back to the mission of the event

Example: “XXX, I’d expect an anti-gun advocate to be more collected. if you are truly trying to win over any readers to be anti-gun advocates perhaps leave out the personal slams. I do hope you read your messages to XXX, and sincerely apologize to her. In my humble opinion it was completely unwarranted. Have a lovely evening.” [Facebook user]

**Comments by Host**

Any comment from Seattle Children’s Hospital

- Seattle Children’s Hospital outreach comment or response to an inquiry

Example: “We have a couple hundred items to give away, XXX. If you can’t come to the giveaway Saturday, visit www.lokitup.org for retailers in King County who are offering a discount on select storage devices or lock boxes through December 2014.” [Facebook user]

**Spam**

Anything unrelated to the topic of firearm safety, Seattle Children’s Hospital, or the event

### Aim 2: Quantitative Analysis for Responsiveness

#### Prevalence and Reach

Among the 13 events for which comments were coded, 4 did not yield data from SimplyMeasured to allow for positive and negative feedback prevalence to be calculated. Among the 9 events with this type of data, the total reach on Facebook was 491,155 (range 10,536-187,072).

#### Positive Feedback

The average number of likes was 1271.3 per event, with the highest of 13.63 per 1000 engagements. Engagements were defined as the average number of likes, comments, and shares on all posts published during the reporting period. The average number of clicks per event was 5183.6 for all events. The range of clicks was 18.0-76.5 per 1000 engagements.

#### Negative Feedback

The average count of times in which “hide post” was clicked on the event posts was 72.3, equating to 1.3 counts per 1000 engagements. The highest prevalence of such clicks was 5.8 per 1000 engagements and the lowest was 0.41 counts per 1000 engagements. There were no posts reported as spam. The average count of “hiding all future posts” was 8.6, equating to 0.16 counts per 1000 engagements. Among all events, the highest prevalence of “hiding all future posts” was 0.88 per 1000 engagements, whereas the lowest was 0.1 per 1000 engagements. There were only 2 counts of “unliking” the page among the 9 events, and both instances happened on the same event post.

### Aim 3: Quantitative Survey Data

Although there were 15 events that occurred during the timeframe of interest, two events did not use the intake survey. Owing to lack of data for these two events, these two event locations were not included in this portion of the analysis. One of the locations missing survey data was for an event that was held in a city in a large urban county (RUCC=1). The second event without survey data was located in a city in a small semiurban county (RUCC =3).

There were two versions of the paper survey: one for the first 3 events and one for the subsequent 10 events. The question regarding location of residence was assessed in the second version of the survey, but not in the first. Owing to the lack of data for the first three events and high levels of missing data, this question was not incorporated in the analysis. There were overlapping questions between the two surveys (see Multimedia Appendix 1 for the sample survey). The questions that overlapped across the two versions of the survey were used. For the purposes of this analysis, similar question answers were aggregated.

Only data with completed event location information and marked as “complete” by the data importer were used for this evaluation. Among the 5512 survey records found in the survey database, only 4054 (81.7%) were marked as “complete” by the data importer, and included complete data on how the participant found out about the event and the location of the event.

Based on the question inquiring how the participant found out about the giveaway event, 1457 (35.9%) indicated some form of social media. Alternatively, 2597 (64.1%) participants indicated that they learned about the event through the newspaper, a friend, the radio, a flyer, word of mouth, working at the store, came to the store at the time of the event, or through other means. See Table 2 for more details on the distribution of how participants learned of these events.

**Table 2:** Proportion of participants’ responses as to how they learned about Safe Firearm Storage Giveaway Outreach Events (SFSGOE) 2014-2018 based on the intake survey.

How did you learn of these SFSGOE?^a^	Responses, n (%)^b^
Social media^c^	1457 (34.6)
Newspaper	383 (9.1)
Friend	329 (7.8)
Radio^d^	41 (1.0)
(Event) Flyer	261 (6.2)
Word of mouth^e^	638 (15.1)
Work at the store^e^	37 (0.9)
Came to the store when the event was occurring^e^	411 (9.7)
Other	660 (15.7)

^a^Participants were asked to “mark all that apply”; therefore, the total of these responses (N=4217) exceeds the number of total participants.

^b^Based on the total number of responses.

^c^With two versions of the survey, the version used for the last 10 events included distinguishing whether the participant found out about the event through Facebook or Twitter. These data were aggregated given the small number of participants who reported Twitter (<1.0%).

^d^With two versions of the survey, the version used for the first three events included radio as a potential response that was not included as an option for respondents to the second version of the survey. This option may have been answered as “other” for participants who used the second version of the survey.

^e^With two versions of the survey, the version used for the last 10 events included these responses that were not included as options for participants who responded to the earlier version of the survey. These options may have been answered as “other” for participants who used the earlier version of the survey.

With respect to the distribution of participant data across locations, 93.2% (3778/4054) of participants with a completed survey were attendees of an urban-located event. Out of all participants, 6.8% (276/4054) with a completed survey were attendees of a semirural-located event. The number of completed surveys for these events ranged from 161 to 406. The event with the least number of completed surveys was the event held in November of 2015 (Kirkland) and that with the largest number of completed surveys was the event held in January of 2015 (Fife). Due to the skew of data toward urban event participants, we were unable to find results that were statistically significant in comparing how participants found out about the event and the location of the event (*P*=.08).

Table 3 compares the characteristics of participants who learned of the event through social media with those of participants who learned about the event through other means. On average, participants who learned about the event via social media were 6.4 years (95% CI 5.5-7.3) younger than those who learned about the event via other means.

In both groups, more participants identified as male. A slightly lower proportion of those who learned about the event via social media indicated that they were not current users of a gun storage/safety device (*P*=.30).

A high intention to use was identified based on survey responses for the giveaway firearm storage device for all event participants, regardless of how they found out about the events; however, the intention to use was significantly higher among the social media group than the nonsocial media group (*P=*.001).

**Table 3:** Characteristics of participants who found out about Safe Firearm Storage Giveaway Outreach Events (2014-2018) through social media or other means.

Participant characteristic/survey response		Social media (n=1457)	Other means (n=2597)
Age (years), mean (SD)		38.8 (11.8)	45.2 (15.5)
**Age category (years), n (%)^a^**			
	18-29	311 (22.8)	410 (18.0)
	30-39	500 (36.7)	539 (23.7)
	40-49	286 (21.0)	422 (18.5)
	50-59	170 (12.5)	426 (18.7)
	60-69	84 (6.2)	338 (14.8)
	≥70	11 (0.8)	143 (6.3)
**Gender, n (%)^b^**			
	Male	801 (55.9)	1553 (64.2)
	Female	629 (43.9)	860 (35.5)
	Other	3 (0.2)	7 (0.3)
**Currently use gun storage device^c^, n (%)**			
	Yes (gun safe, gun lock box, trigger lock, cable lock, other)	1015 (70.5)	1698 (68.7)
	No	424 (29.5)	772 (31.3)
**Currently store gun(s) loaded^d^, n (%)**			
	Yes, all of them	428 (30.0)	568 (23.4)
	Yes, some of them (some are and some aren’t)	320 (22.3)	536 (22.1)
	None of them	578 (40.3)	1078 (44.4)
	Not sure	15 (1.0)	36 (1.5)
	Does not apply to my home	93 (6.5)	211 (8.7)
**Intend to use gun safety device in the next week^e^, n (%)**			
	Yes	1331 (92.2)	2201 (89.1)
	No	39 (2.7)	119 (4.8)
	Unsure/not sure	73 (5.1)	151 (6.1)
**RUCC^f^ based on location of event^g^, n (%)**			
	1: urban (large county)	783 (53.7)	1291 (49.7)
	2: urban (medium county)	412 (28.3)	701 (27.0)
	3: urban (small county)	149 (10.2)	442 (17.0)
	4: semirural (large county)	0 (0.0)	0 (0.0)
	5: semirural (medium-large county)	113 (7.8)	163 (6.3)

^a^Due to missing answers, 1362 and 2278 participants responded to this question in the social media and nonsocial media group, respectively.

^b^Due to missing answers, 1433 and 2420 participants responded to this question in the social media and nonsocial media group, respectively.

^c^Due to missing answers, 1439 and 2470 participants responded to this question in the social media and nonsocial media group, respectively. For this question, participants were asked to “mark all that apply”; therefore, the total number of recorded responses (N=4925) is larger than the number of participants. The option “other” was added to the second version of the survey. These data represent aggregated responses for indication of use of any of the described firearm storage device and “other.”

^d^Due to missing answers, 1434 and 2429 participants responded to this question in the social media and nonsocial media group, respectively. This question and the answer options were different for the two versions of the survey. The second version added the option of “not sure” and “does not apply to my home.”

^e^Due to missing answers, 1443 and 2471 participants responded to this question in the social media and nonsocial media group, respectively.

^f^RUCC: United States Department of Agriculture Economic Research Service Rural-Urban Continuum; codes from 2013 were used to categorize locations (refer to Table 1). There were no events held and included in this analysis for RUCC 6, 7, 8 and 9.

^g^Due to missing answers, 1457 and 2597 participants responded to this question in the social media and nonsocial media group, respectively.

## Discussion

### Principal Findings

To our knowledge, this study is one of the first to investigate the receptiveness and responsiveness of using social media as a marketing tool for a safe firearm storage giveaway intervention. Prior to this study, it was unknown whether or not there would be receptiveness and responsiveness in using social media to market these events. Much of this uncertainty stemmed from acknowledging the controversial nature of this topic and the need to assess whether there would be acceptance of using this tool. Additionally, if there was acceptance, it was unclear whether the level of acceptance would differ between rural and urban areas.

Including the two terms of “receptiveness” and “responsiveness” to conceptualize the aims of the study helped with conciseness, and encapsulated the goals of health and social media research, especially with the public-facing metrics and data available on Facebook. As these terms are generally defined, they can be expanded upon and used by other social media researchers when trying to understand the acceptability and reach of health promotion campaigns for other social media platforms as well.

We found high levels of acceptability and positive receptiveness toward these social media posts through user comments. The content analysis showed a higher proportion of positive than negative comments. Facebook generated an avenue for users to not only find out about the event but also to share the event through easily “tagging” other Facebook users in the comment. Individuals providing comments asking for more from these events were specifically geared toward having the events closer to where they reside, indicating that there is a call and need for these events.

Promoting the events on Facebook also allowed for conversations between users and Seattle Children’s Hospital, the host of the events. Facebook users were able to comment with questions about the event and either other users or the host could respond in a more immediate manner. Findings from this study also support the presence of certain non-host Facebook users or “moderators,” who speak up to draw the conversations back to the goal of the event (ie, to protect children and households through safe firearm storage practices). Moreover, these individuals facilitate the conversation in a direction that deters the discussion away from topics such as the legalities of firearm ownership.

Although there were some negative and advocacy-oriented comments, there was an emergence of users who implicitly function as moderators on Facebook along with ability for the host to respond and moderate as well. Moderators have the potential to also lift some of the burdens off the event host to have to constantly monitor and intervene. Previous studies have found that moderators in online communities have a substantial contribution in keeping discussions on track by countering negative social media perceptions [25]. This observation begins to highlight the emerging importance of engaging positive influencers on social media for promotion of events such as the SFSGOE. Other studies have also highlighted the importance of having “moderated” discussions, which have been shown to yield better participant engagement and to promote a safer discussion environment [26,27].

We also observed a much higher level of positive feedback than negative feedback. There were more interactions such as clicking and liking the post compared to hiding or unliking the post. This high level of positive responsiveness is important because it shows that despite the controversial subject matter, no notable backlash toward the organization hosting the event or any perceivable impact on their clinically oriented branding occurred.

Over one-third of all participants across the 13 events found out about the event through social media, indicating that this is a useful marketing tool. Additionally, more of the younger participants and those who identified as female found out about these events through social media than through the other promotion tools such as flyers, newspapers, and word of mouth. Those who found out about the event on social media were on average 6.4 years younger than those who found out through other means. Contextually, this represents the users of Facebook, in which there is a higher proportion of female users compared to male users (74% vs 62% of US adults) and a decreasing trend in usage with increasing age [28]. This is important when considering the intended demographic characteristics of reach.

There was no significant difference in current firearm storage practices between the social media group and the other group. Participants who found out about the event on social media expressed an even greater intention to use the giveaway event storage device than those who found out about the event through other means. When implementing an intervention, it is important to use the most appropriate and efficient tools to promote it. This study suggests that social media captures younger individuals and a different gender distribution than means such as flyers, newspaper ads, and posters. This finding further supports other social media and health promotion studies that have also found social media to be an acceptable communication tool; however, the effectiveness varies across different demographic groups [29,30].

Using a concurrent mixed methods approach, the integration of the qualitative and quantitative methodology allowed for results from both arms of the study to complement and support each other. The social media analysis allowed for gaining an understanding of receptiveness and responsiveness, and we were further able to use the data from the survey to understand the type of people that social media promotions reached and the characteristics of that population.

However, due to the limited qualitative and quantitative data for rural locations, we were unable to determine whether social media would be able to improve the reach in marketing safe firearm storage promotion events.

### Limitations

In using Facebook data to understand receptiveness, there are likely to be biases in the attitudes presented. Across social media platforms, including Facebook, there is a tendency for greater positive expressions because they are generally perceived as more appropriate than negative sentiments [31]. Similarly, we found many more positive comments than negative comments. Facebook users with negative perceptions toward firearm storage may not find Facebook to be the most appropriate platform to express that sentiment, since it is a publicly facing platform and also amidst comments that lean more heavily toward the positive. Additionally, there is the chance that certain comments were deleted or reported by Facebook users by the time we were able to retrospectively export data for this analysis. To our knowledge, no comments were removed by Seattle Children’s Hospital.

Due to the finite number of comments publicly available for this exploratory content analysis, we have limited understanding of the saturation of these codes. With the inductive thematic saturation approach, we were able to generate seven themes; however, future studies with greater elements of coding would allow gaining a better understanding of the reproducibility of use in this exploratory inductively generated codebook [22].

The social media content analysis evaluated data from 13 events, although 15 events occurred between December 2014 and March 2018. There were two events that were not evaluated because they were not publicly available on Facebook at the time of data extraction. This decreased the sample size for our qualitative analysis and also may have decreased the generalizability of the results due to the homogeneity of the settings. Of the 13 events evaluated qualitatively, 8 were held in larger cities from urban counties and only 1 was located in a city from a semirural county. Additionally, through the quantitative portions of the social media evaluation, an additional 4 events did not yield data through SimplyMeasured, the social media analytics software. Because of these missing data, there may have been a loss in positive feedback data as well as negative feedback data. Given this level of missing social media data, the findings described in this study may only be generalizable to events held in urban areas.

Additionally, of the 5512 records of surveys collected, there was an 81.7% (4504/5512) completion response rate. We defined a complete survey as one with location of the event attended indicated and that was marked as “complete” by the data entry program staff. There could be a systematic difference between those who completed the survey and those who chose not to complete any or certain fields of the survey. Moreover, with the analyzed data, we found much higher levels of missingness among respondents who indicated that they found out about the events through means other than social media. Dependent on the characteristic of interest, the proportion of missing data ranged from 4.9% to 12.2%. An added limitation of the results from the survey analysis is that, due to its anonymous nature, it is possible that participants could have attended more than one event; therefore, their characteristics would have been counted more than once during aggregate analysis. We did not have identifiers to disaggregate and remove any repeated responses from the same participant.

For the purposes of this study, we used the location of the event as a proxy to generally understand if the participants were from rural or urban areas. However, we were unable to truly ascertain the city or county that the participants were traveling from to attend these events. This was because location of residence was not asked in the survey for the earlier events, and there were very high levels of missing data from the surveys completed in the latter events. Obtaining information about where participants are coming from would have better informed not only how far the participants were traveling but also how social media marketing may be reaching communities closer to or further from the location of these events.

### Conclusions

We found high levels of positive receptiveness and responsiveness toward event posts promoting SFSGOE. Specifically, these social media posts reached a greater proportion of younger participants. Among participants who identified as female, a greater proportion found out about these events through social media. Due to missing data and stronger data skew for urban area events, we were unable to draw conclusions as to whether or not there were differences in acceptability of such events or using social media to market these events between rural and urban areas.

Social media allowed for multifaceted interactions from user to user, from the user to the host, and from the user to the social media platform. We also found the emergence of Facebook “moderators” whose implicit role helped in supporting the goals of the health promotion event. These interactions are unique because social media facilitate more immediate displays of receptiveness and responsiveness compared with more traditional methods of health promotion, such as posters and flyers. Our analysis showed high levels of acceptability in using social media to market an intervention that may have more controversial connotations. Additionally, our findings support the idea that social media can be used as a tool for health promotion, specifically as a tool for promoting and discussing community-focused interventions. Future research should focus on understanding if there is a difference in rural vs urban acceptability for firearm storage education and promotion, and on the greater implementation of these events to encourage safe firearm storage practices. Additionally, further research can expand the understanding of implementing health promotion activities on social media platforms beyond Facebook, such as on Twitter and Instagram.

## Appendix

Multimedia Appendix 1 SFSGOE Intake Survey.

## Abbreviations

RUCC: Rural-Urban Continuum Code
SFSGOE: Safe Firearm Storage Giveaway Outreach Event
