# Supplementary material for: Receptiveness and Responsiveness Toward Using Social Media for Safe Firearm Storage Outreach: Mixed Methods Study
Source: J Med Internet Res. 2021 Jun 18;23(6):e24458. doi: 10.2196/24458 (PMC8277364; doi:10.2196/24458)
Supplement: Multimedia Appendix 1 [file jmir_v23i6e24458_app1.pdf]

# Firearm Safety Event Survey

## *Please Circle Your Answers:*

1. **How did you know about today's event?** *(circle all that apply)*
   a. Facebook
   b. Twitter
   c. Newspaper
   d. Event flyer
   e. I work at the store
   f. Word of mouth
   g. I came to the store
   h. Other: _____________________

2. **Do you think that this was a good place to have this event?**
   a. Yes
   b. Not sure
   c. No

3. **Where else should we hold events?**

   ______________________________________

4. **What's the *main reason* you want a gun safety device?**
   a. Overall gun safety
   b. To keep kids safe
   c. Store other valuables (not guns)
   d. Protect guns from theft
   e. Gift for someone
   f. Other: ______________________________

   ____________________________________

5. **Which safety device would you prefer to receive today?**
   a. Lock box
   b. Trigger lock
   c. No preference
   d. Only here for information and do not plan to receive a device

6. **Do you plan to use this safety device to store your gun(s) within the next week?**
   a. Yes
   b. No
   c. Not sure

7. **What kind of gun safety device do you currently use?** *(circle all that apply)*
   a. None
   b. Gun safe
   c. Gun lock box
   d. Cable lock
   e. Trigger lock
   f. Other: _____________________

8. **Are the guns in your home currently stored in a locked place?** *(Examples include using a locked cabinet, locked box, trigger lock or cable gun lock)*
   a. Yes, all of them
   b. Yes, some of them
   c. None of them
   d. Not sure
   e. No guns are in my home

9. **Are the guns in your home currently stored loaded?**
   a. Yes, all of them
   b. Yes, some of them
   c. None of them
   d. Not sure
   e. No guns are in my home

10. **Is the ammunition in your home currently stored in a locked place?**
    a. Yes, all of it
    b. Yes, some of it
    c. None of it
    d. Not sure
    e. No ammunition is in my home

11. **Who lives in your household?** *(circle all the apply)*
    a. Spouse or significant other
    b. Children *10 years old or younger*
    c. Children *older than 10 years of age*

12. **What is your gender?**
    a. Male    b. Female    c. Other

13. **What is your age?** _______________________

---

***We would greatly appreciate any other comments you have!***

______________________________________________________________

______________________________________________________________

***Thank you for your participation today!***
